# Occurrence of the Colistin Resistance Gene *mcr-1* and Additional Antibiotic Resistance Genes in ESBL/AmpC-Producing Escherichia coli from Poultry in Lebanon: A Nationwide Survey

**DOI:** 10.1128/Spectrum.00025-21

**Published:** 2021-09-08

**Authors:** Myriam Mikhayel, Sébastien O. Leclercq, Dolla Karam Sarkis, Benoît Doublet

**Affiliations:** a ISP, Institut National de Recherche pour l’Agriculture, l’Alimentation, l’Environnement, Université de Tours, Nouzilly, France; b Laboratoire des Agents Pathogènes, Faculté de Pharmacie, Université Saint Joseph de Beyrouth, Beirut, Lebanon; c Cogitamus Laboratory, Nouzilly, France; University of Illinois at Urbana Champaign

**Keywords:** multidrug resistance, CTX-M-3, poultry, CMY-2, antibiotic usage

## Abstract

The objective of the present study was to determine genomic characteristics of expanded-spectrum cephalosporin (ESC)-resistant Escherichia coli spreading in healthy broilers in Lebanon in 2018. Rectal swabs (*n* = 280) from 56 farms were screened for the presence of ESC-resistant E. coli isolates. Antimicrobial susceptibility and extended-spectrum β-lactamase (ESBL)/AmpC production were determined by the disk diffusion method. Whole-genome sequencing (WGS) of 102 representative isolates of E. coli was performed to determine their phylogenetic diversity, serotypes, sequence types (ST), acquired resistance genes, and virulence-associated genes. Fifty-two out of 56 farms housed broilers carrying ESC-resistant E. coli isolates. These farms had large and recurrent antimicrobial practices, using, for some of them, critically important antibiotics for prophylactic and therapeutic purposes. Among the 102 sequenced multidrug-resistant (MDR) E. coli isolates, the proportion of ESBL, plasmid-mediated AmpC β-lactamase (pAmpC) producers, and ESBL/pAmpC coproducers was 60%, 27.6%, and 12.4%, respectively. The most prevalent ESBL/pAmpC genes were *bla*_CMY-2_, *bla*_CTX-M-3_, *bla*_CTX-M-15_, *bla*_CTX-M-27_, and *bla*_CTX-M-14b_ (*n* = 42, *n* = 31, *n* =15, *n* = 9, and *n* = 7, respectively). These ESBL/pAmpC producers were distributed in different STs, most being well-known avian-associated and sometimes pathogenic STs (ST-10, ST-48, ST-93, ST-115, ST-117, and ST-457). Phylogenetic single nucleotide polymorphism (SNP) analysis confirmed their genetic diversity and wide dispersion across the Lebanese territory. Most isolates were also resistant to ciprofloxacin (101/102 with 3 QRDR mutations), and 19/102 isolates from 11 unrelated STs also carried the mobile resistance gene *mcr-1*. This survey illustrates the alarming prevalence of MDR E. coli resistant to medically important antibiotics in broilers in Lebanon. This advocates the need for surveillance programs of antimicrobial resistance in Lebanon and the reduction of excessive use of antibiotics to limit the spread of MDR E. coli in food-producing animals.

**IMPORTANCE** Poultry production is a main contributor of the global trend of antimicrobial resistance arising from food-producing animals worldwide. In Lebanon, inappropriate use of antibiotics is frequent in chickens for prophylactic reasons and to improve productivity, resulting in an alarming prevalence of extended-spectrum β-lactamase (ESBL)/AmpC-producing Escherichia coli, also resistant to other medically important antibiotics (i.e., colistin and ciprofloxacin). Their complex genomic epidemiology highlighted by an important genetic diversity suggests that these resistance determinants are largely spreading in enteric bacteria in Lebanese poultry. Further molecular surveillance is needed to understand the country-specific epidemiology of ESBL/AmpC and *mcr-1* genes in Lebanese poultry production. In addition, decisive interventions are urgently needed in order to ban the use of critically important antibiotics for human medicine in food-producing animals and limit the spread of antibiotic resistance in Lebanon.

## INTRODUCTION

Antimicrobial resistance (AMR) is a serious public health problem worldwide in both humans and animals. The main driver for the evolution of bacterial resistance is the excessive usage of antibiotics in human and veterinary medicine. In some countries, antibiotics remain heavily administered for therapeutic and prophylactic purposes in veterinary medicine. Moreover, there is an increasing public health concern about zoonotic transmission of resistance to critically important antimicrobial classes, such as expanded-spectrum cephalosporins (ESC), carbapenems, fluoroquinolones, and colistin (1). Although the extent to which food of animal origin contributes to the zoonotic transmission of multidrug-resistant (MDR) bacteria remains an ongoing debate, the transfer of enteric resistant strains from livestock to humans through the handling and consumption of undercooked animal meats is known to occur (2). During the two last decades, the prevalence of extended-spectrum β-lactamase (ESBL)- and plasmid-mediated AmpC β-lactamase (pAmpC)-producing Gram-negative bacteria has become extensively reported in food-producing animals (3, 4). Of particular concern is the high prevalence of ESBL/pAmpC-producing *Enterobacterales* in intensive broiler production around the world (5). The intestinal microbiome of these animals might serve as a reservoir for ESBL/pAmpC-producing strains capable of being transmitted to humans. The epidemiology of ESBL/AmpC-encoding genes is rather complex, since a huge variety of different *bla*_ESBL/AmpC_ genes and gene families exist and spread on various mobile genetic elements such as plasmids and integrative conjugative elements that are able to transfer horizontally between bacterial strains and species. Furthermore, plasmids carrying *bla*_ESBL/AmpC_ genes may also persist in bacterial clones of defined sequence types (ST), spreading geographically and in different hosts (4).

Since the discovery of plasmid-mediated colistin resistance by the *mcr-1* gene in human and animal isolates in China in 2015, several studies have reported an increasing trend of coexistence of *mcr* genes and ESBL/AmpC genes in the same bacterial isolates (6). Worldwide, there are more reports of *mcr*-mediated colistin resistance in food-producing animals, notably in poultry, than in humans, which suggests that farm animals are a reservoir of multiple critically important resistances (6). Moreover, *mcr-1*-positive and/or ESBL/pAmpC-producing Escherichia coli from healthy broilers were usually classified as commensal strains, but recent investigations found that among these resistant strains, some also harbor several of the genetic virulence factors of avian pathogenic E. coli (APEC) or extraintestinal pathogenic E. coli-like (ExPEC) strains and, therefore, have the ability for an enhanced colonization of the gut (7).

Lebanon is a country with developing antimicrobial stewardship and AMR surveillance programs, in which several studies have been conducted to address the occurrence of AMR in clinical human settings at the phenotypic level (8). However, large-scale genomic studies addressing AMR in humans or in food-producing animals remain scarce (2). Different studies have recently described the occurrence of ESBL-producing or *mcr-1*-positive *Enterobacteriaceae* in livestock or in environmental water sources, but none of them have performed a nationwide genomic analysis of resistant bacteria (for review, see reference 2 and references 9–15).

The aim of the present study was to assess the occurrence of ESBL/AmpC-producing *Enterobacterales* in different Lebanese broiler farms. In addition, a questionnaire was directed to the veterinarians and farmers to record rearing practices and antibiotic usage at the farm level. Then, the objective was to characterize ESBL/AmpC E. coli isolates at the phenotypic level and by whole-genome sequencing to analyze their genetic diversity, resistance and virulence gene content, epidemiology, and dissemination in the Lebanese poultry production.

## RESULTS

### Sampling and antibiotic usage.

**FIG 1:**
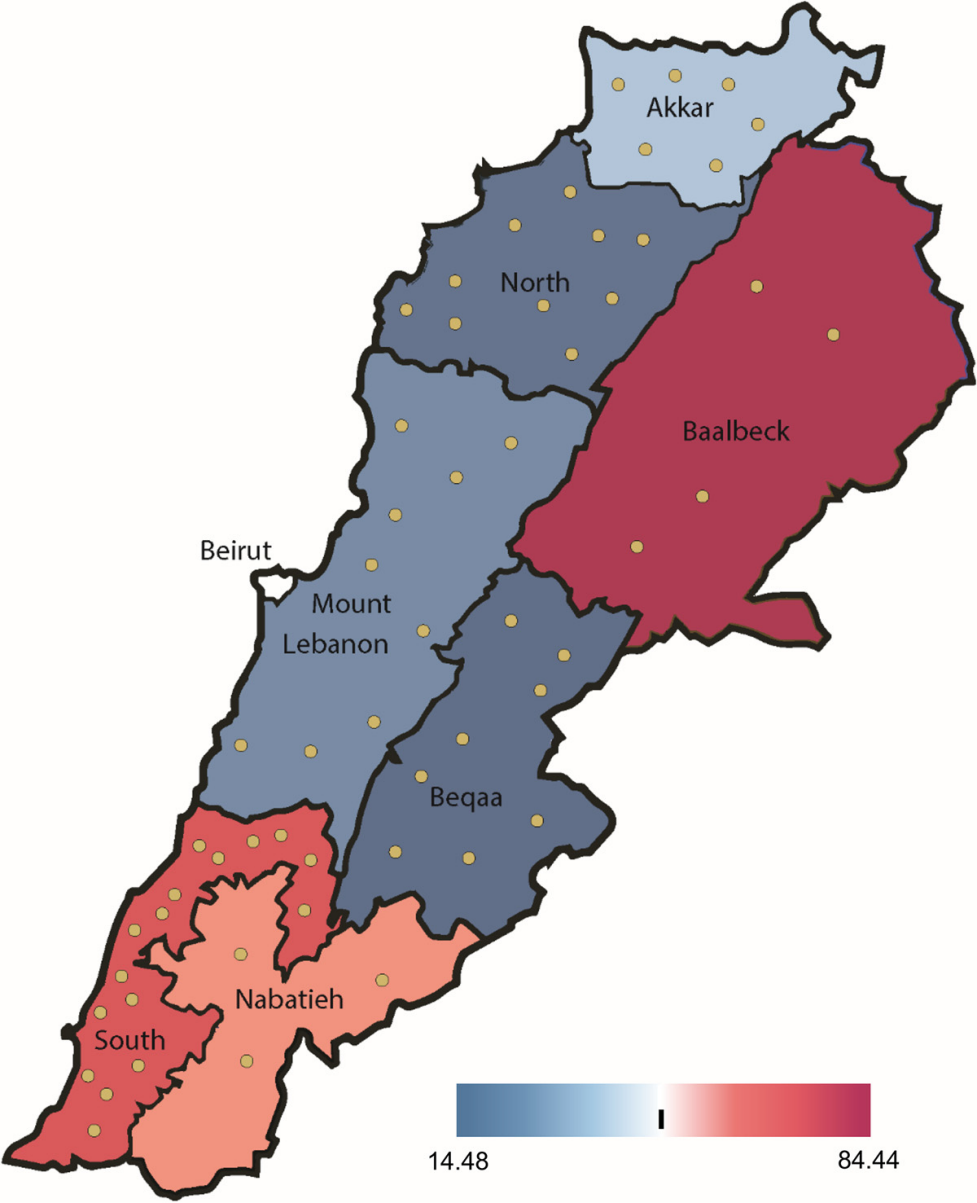
Antimicrobial treatment incidence in broiler farms for each administrative region in Lebanon. Antimicrobial usage was quantified using the number of active substances administered multiplied by treatment duration (days) divided by the number of farms as a proxy of treatment incidence for a region. The administrative regions are colored according to their treatment incidence value. Yellow circles represent farm locations.

A total of 280 individual fecal samples were collected from healthy broiler flocks (5 birds/flock; birds from 12 to 45 days old) during summer and fall 2018 in 56 selected farms representing the main regions of poultry production in Lebanon. Farm rearing practices were collected through a questionnaire, and answers are summarized in Table S1 in the supplemental material. Antimicrobial treatment data revealed that all farms except 4 located in South (*n* = 3) and Mount Lebanon (*n* = 1) are systemically using antibiotics for prophylactic purposes. Depending on farms, antimicrobial treatments were applied at the chick’s arrival but differed in duration from 3 to 30 days and in the number of antibiotics administered, from doxycycline alone to up to 8 different antibiotics (Table 1). Antimicrobial usage was summarized by an overall treatment incidence at the region level, which indicates that the highest usage of antibiotics is in the farms of the region of Baalbeck, followed by the farms of the southern area of Lebanon (Fig. 1). Several critically important antibiotics of the top-priority list of WHO are used in broilers, such as colistin and ciprofloxacin in 62.5% and 50% of sampled farms, respectively, as well as enrofloxacin, cefpodoxime, and several large-spectrum macrolides (Table 1; Fig. S1). Other antibiotic families were also largely used, such as tetracyclines (doxycycline) and aminoglycosides (gentamicin) (Fig. S1).

**TABLE 1:** Antibiotic treatments in the sampled farms

Farm ID^*a*^	Region	Administered antibiotics	Antibiotic treatment duration (days)	Reasons for antibiotic usage
A1	Akkar	Amoxicillin, ampicillin, cephalexin, ciprofloxacin, colistin, doxycycline, gentamicin, trimethoprim/sulfamethoxazole	6	Prevention and cure
A2	Akkar	Amoxicillin, ampicillin, cephalexin, ciprofloxacin, colistin, doxycycline, gentamicin	6	Prevention and cure
A3	Akkar	Amoxicillin, ampicillin, cephalexin, ciprofloxacin, colistin, doxycycline, florfenicol, tilmicosin	3	Prevention and cure
A4	Akkar	Ciprofloxacin, colistin, doxycycline, florfenicol, gentamicin	6	Prevention and cure
A5	Akkar	Colistin, doxycycline, tilmicosin	6	Prevention and cure
A6	Akkar	Ciprofloxacin, colistin, doxycycline, tilmicosin	5	Prevention and cure
Bb1	Baalbeck	Colistin, doxycycline, florfenicol, gentamicin	10	Prevention and cure
Bb2	Baalbeck	Colistin, doxycycline, florfenicol, gentamicin	10	Prevention and cure
Bb3	Baalbeck	Ciprofloxacin, colistin, doxycycline, trimethoprim/sulfamethoxazole	30	Prevention and cure
Bb4	Baalbeck	Ciprofloxacin, colistin, doxycycline, trimethoprim/sulfamethoxazole	30	Prevention and cure
B1	Beqaa	Colistin, doxycycline, florfenicol, gentamicin, tilmicosin	10	Prevention and cure
B2	Beqaa	Ciprofloxacin, doxycycline, gentamicin	10	Prevention and cure
B3	Beqaa	Amoxicillin, doxycycline, gentamicin	5	Prevention and cure
B4	Beqaa	Ciprofloxacin, doxycycline	10	Prevention and cure
B5	Beqaa	Doxycycline	5	Prevention and cure
B6	Beqaa	Amoxicillin, ampicillin, cephalexin, ciprofloxacin, colistin, doxycycline, gentamicin, trimethoprim/sulfamethoxazole	10	Prevention and cure
B7	Beqaa	Ciprofloxacin, doxycycline, gentamicin, trimethoprim/sulfamethoxazole	5	Prevention and cure
B8	Beqaa	Doxycycline	5	Prevention and cure
M1	Mount Lebanon	Doxycycline, florfenicol, gentamicin, tetracycline, trimethoprim/sulfamethoxazole	5	Prevention and cure
M2	Mount Lebanon	Ciprofloxacin, doxycycline, gentamicin	3	Prevention and cure
M3	Mount Lebanon	Ciprofloxacin, doxycycline	5	Prevention and cure
M4	Mount Lebanon	Ciprofloxacin, doxycycline, gentamicin	5	Prevention and cure
M5	Mount Lebanon	Ciprofloxacin, tilmicosin	5	Prevention and cure
M6	Mount Lebanon	Cephalexin, ciprofloxacin, colistin, doxycycline, gentamicin, trimethoprim/sulfamethoxazole	3	Prevention and cure
M7^*b*^	Mount Lebanon	NA^*c*^	NA^*c*^	Cure (only in case of infection)
M8	Mount Lebanon	Ciprofloxacin, doxycycline, gentamicin, trimethoprim/sulfamethoxazole	10	Prevention and cure
M9	Mount Lebanon	Ciprofloxacin, doxycycline, gentamicin, trimethoprim/sulfamethoxazole	10	Prevention and cure
N1	Nabatieh	Amoxicillin, colistin, doxycycline, enrofloxacin, gentamicin, tilmicosin, trimethoprim/sulfamethoxazole	10	Prevention and cure
N2	Nabatieh	Amoxicillin, colistin, doxycycline, enrofloxacin, gentamicin, tilmicosin, trimethoprim/sulfamethoxazole	10	Prevention and cure
N3	Nabatieh	Amoxicillin, colistin, doxycycline, enrofloxacin, gentamicin, tilmicosin, trimethoprim/sulfamethoxazole	15	Prevention and cure
ND1	North	Cephalexin, doxycycline, gentamicin	3	Prevention and cure
ND2	North	Cephalexin, doxycycline, gentamicin	3	Prevention and cure
ND3	North	Cephalexin, doxycycline, gentamicin	3	Prevention and cure
ND4	North	Ciprofloxacin	3	Prevention and cure
ND5	North	Doxycycline	5	Prevention and cure
ND6	North	Doxycycline	5	Prevention and cure
ND7	North	Doxycycline	5	Prevention and cure
ND8	North	Ciprofloxacin, colistin, doxycycline, gentamicin	6	Prevention and cure
ND9	North	Ciprofloxacin, colistin, doxycycline, gentamicin, trimethoprim/sulfamethoxazole	10	Prevention and cure
ND10	North	Ciprofloxacin, colistin, doxycycline, gentamicin, tilmicosin	6	Prevention and cure
S1	South	Amoxicillin, colistin	10	Prevention and cure
S2	South	Amoxicillin, colistin	10	Prevention and cure
S3	South	Amoxicillin, colistin, tilmicosin	30	Prevention and cure
S4	South	Amoxicillin, colistin, erythromycin, neomycin	15	Prevention and cure
S5	South	Amoxicillin, colistin, erythromycin, neomycin	15	Prevention and cure
S6	South	Amoxicillin, colistin, erythromycin, neomycin	15	Prevention and cure
S7	South	Colistin, enrofloxacin, gentamicin, sulfadiazine, trimethoprim/sulfamethoxazole	15	Prevention and cure
S8	South	Amoxicillin, colistin, doxycycline, gentamicin	10	Prevention and cure
S9^*b*^	South	NA^*c*^	NA^*c*^	Cure (only in case of infection)
S10	South	Amoxicillin, colistin, tylosin	30	Prevention and cure
S11	South	Doxycycline, gentamicin	20	Prevention and cure
S12	South	Amoxicillin, cefpodoxime, ciprofloxacin, colistin, doxycycline, gentamicin, spiramycin, tylosin	12	Prevention and cure
S13	South	Amoxicillin, cefpodoxime, ciprofloxacin, colistin, doxycycline, gentamicin, spiramycin, tylosin	25	Prevention and cure
S14^*b*^	South	NA^*c*^	NA^*c*^	Cure (only in case of infection)
S15^*b*^	South	NA^*c*^	NA^*c*^	Cure (only in case of infection)
S16	South	Amoxicillin, cefpodoxime, ciprofloxacin, colistin, doxycycline, gentamicin, spiramycin, tylosin	15	Prevention and cure

a *N* = 56.

b Farms in which no ESC-resistant strains were isolated.

c No antibiotic administration on the sampled flock.

### Selection of ESC-resistant isolates and their antimicrobial susceptibility.

**FIG 2:**
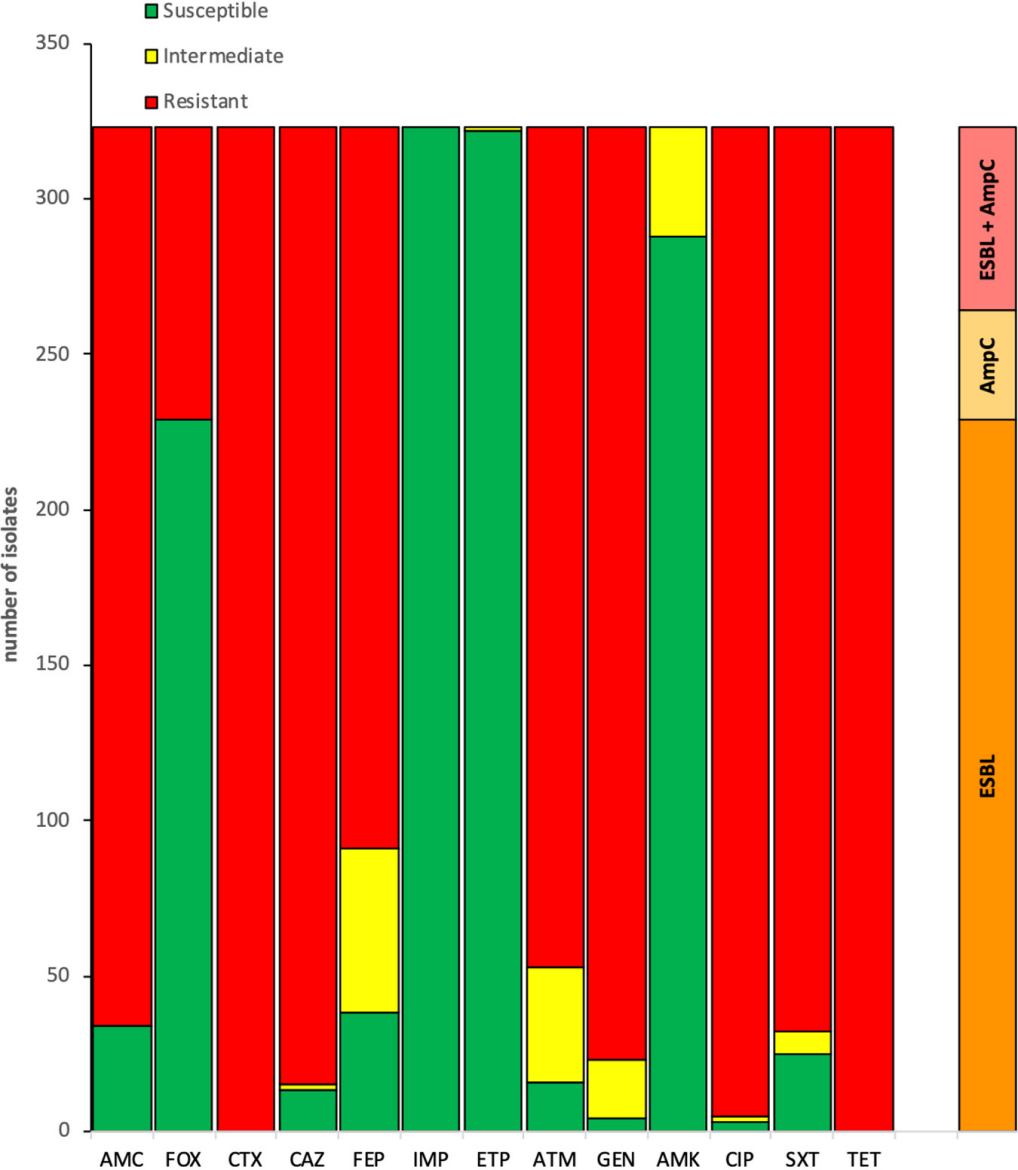
Susceptibility profiles of 323 ESC-resistant *Enterobacterales* isolates and phenotypic ESBL/AmpC detection. AMC, amoxicillin-clavulanate; FOX, cefoxitin; CTX, cefotaxime; CAZ, ceftazidime; FEP, cefepime; IMP, imipenem; ETP, ertapenem; ATM, aztreonam; GEN, gentamicin; AMK, amikacin; CIP, ciprofloxacin; SXT, trimethoprim-sulfamethoxazole; TET, tetracycline.

A total of 323 ESC-resistant isolates were selected from 220 fecal samples originating from 52 farms. No ESC-resistant isolate was found in the 4 farms that did not use antimicrobials for the sampled broiler flocks. One hundred and ninety-two and 131 isolates were selected on cefotaxime and cefepime, respectively. The Escherichia coli species was largely dominant, representing more than 87% (*n* = 283) of the isolates (Table 2; Table S2). Few isolates belonged to the genera Klebsiella, Enterobacter, and Proteus (Table 2). Nonsusceptibility was observed for several antibiotics among the 323 isolates of this ESC-resistant collection (Fig. 2). Indeed, almost all isolates were nonsusceptible to cefotaxime, ciprofloxacin, gentamicin, and tetracyclines. In addition, >89% of these isolates were resistant to amoxicillin-clavulanate and folate inhibitors (trimethoprim-sulfamethoxazole) (Table 2; Fig. 2). All isolates were multidrug resistant (≥3 antimicrobial classes), but all remained susceptible to carbapenems and amikacin. Based on the double disk synergy test and resistance to cefoxitin, 71%, 11%, and 18% of these isolates were suspected to produce ESBL, AmpC cephalosporinases, or both, respectively. None of the breeding practices collected through the questionnaire during sample collection could have been linked to the phenotypic resistance profile by our multiple component analysis (MCA), suggesting that farm-level breeding practice variations have little impact on the resistance profile of circulating *Enterobacterales*.

**TABLE 2:** Proportion of resistance among the 323 ESC-resistant *Enterobacterales* isolates

Antibiotics	E. coli (*n* = 283)	Klebsiella sp. (*n* = 28)	Enterobacter cloacae (*n* = 7)	Proteus mirabilis (*n* = 5)
Amoxicillin-clavulanate	253 (88.8%)	26 (93.1%)	7 (100%)	3 (60%)
Cefoxitin	117 (41.1%)	7 (25%)	0 (0%)	0 (0%)
Cefotaxime	283 (100%)	28 (100%)	7 (100%)	5 (100%)
Ceftazidime	273 (95.8%)	26 (93.1%)	7 (100%)	3 (60%)
Cefepime	201 (70.5%)	23 (79.3%)	6 (85.7%)	3 (60%)
Aztreonam	239 (83.9%)	23 (79.3%)	7 (100%)	3 (60%)
Imipenem	0 (0%)	0 (0%)	0 (0%)	0 (0%)
Ertapenem	0 (0%)	0 (0%)	0 (0%)	0 (0%)
Gentamicin	266 (93.3%)	28 (100%)	3 (42.8%)	5 (100%)
Ciprofloxacin	280 (98.2%)	28 (100%)	7 (100%)	5 (100%)
Trimethoprim Sulfamethoxazole	253 (88.8%)	28 (100%)	7 (100%)	5 (100%)
Tetracyclines	283 (100%)	28 (100%)	7 (100%)	5 (100%)
Amikacin	0 (0%)	0 (0%)	0 (0%)	0 (0%)

### Whole-genome sequencing analysis.

The E. coli species was largely dominant (88%) among this collection of ESC-resistant isolates, and a set of 102 E. coli isolates was selected to be representative of the initial sampling. The 102 assembled draft genomes ranged from 122 to 936 contigs, with sequencing depth ranging from 81× to 235×, a completeness level of >97.5% (mean of 99.5%), and an estimated contamination level of <2.5% (mean of 0.4%). These 102 draft genomes were further analyzed for their phylogenetic diversity, their acquired resistome, and their virulence gene content, as described in the sections below.

### Phylogroup, multilocus sequence typing, and serotyping.

Most of the ESC-resistant E. coli isolates belonged to phylogroup A (46%; *n* = 48), followed by groups E, F, and G (*n* = 14, 12, and 13, respectively). Fewer isolates belonged to phylogroups B1 (*n* = 8), C (*n* = 1), D (*n* = 6), and clade I (*n* = 2), thus confirming the phenotypic identification of these 102 ESC-resistant isolates corresponding as the new species E. coli *sensu lato* (clade I and E. coli being considered two subspecies of a single species) (16).

**FIG 3:**
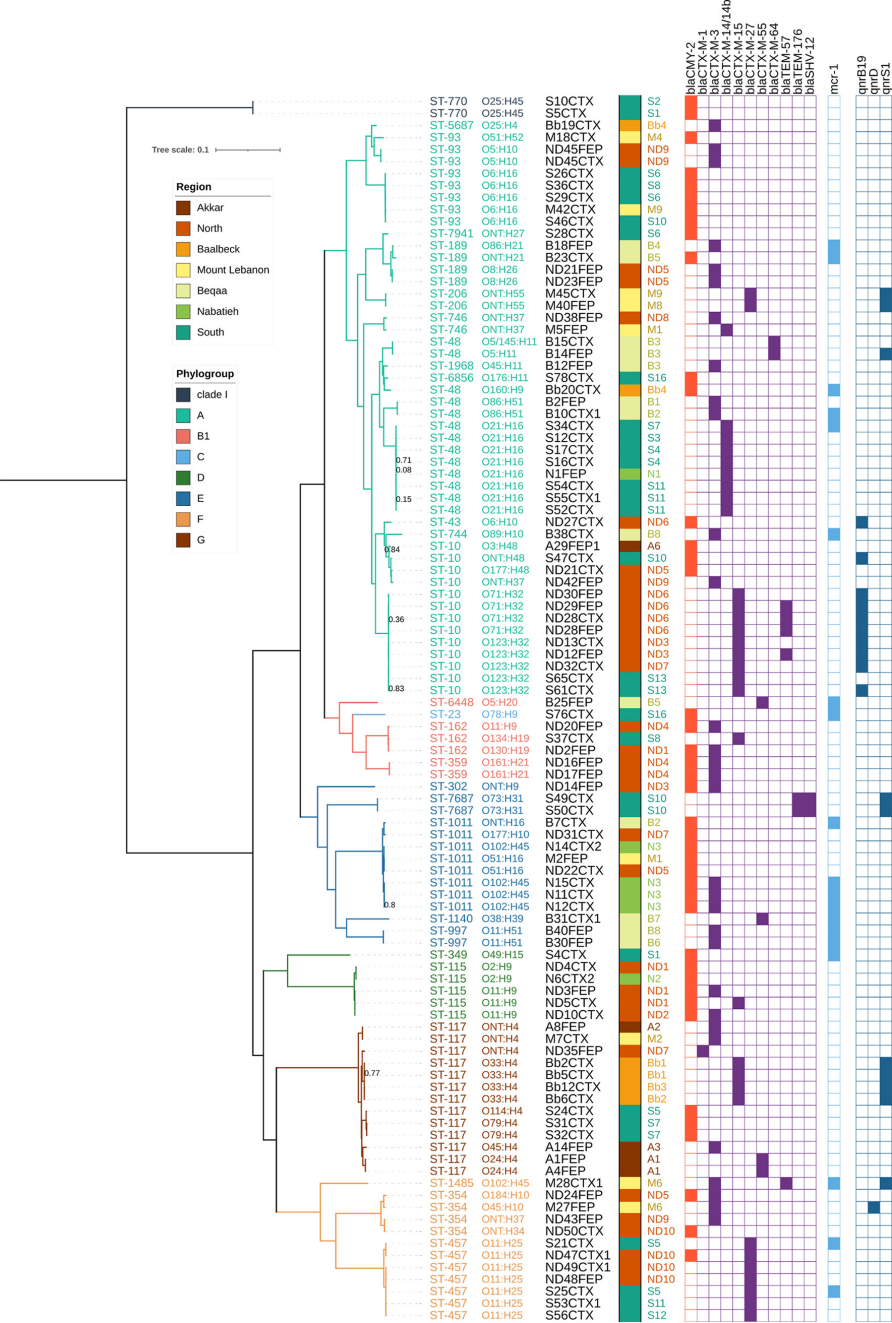
Phylogenetic relationships between the 102 sequenced ESC-resistant E. coli strains. The tree was inferred from the 176,818 SNPs present in genomic regions shared by all isolates. Local branching support values are based on the Shimodaira-Hasegawa test. Values of 1, indicating maximum support, are not displayed. The tree was rooted according to the known relationships between E. coli phylogroups. For each strain, the sequence type, serotype, identifier, region, and farm of origin are indicated in columns 1 to 5 on the right side of the tree. Presence of *bla*_CMY-2_, *bla*_ESBL_ genes, *mcr-1*, and *qnr* resistance genes detected using ResFinder 2.1 are indicated with a filled box. The strain identifier code includes the region initial followed by a unique sampled animal number per region, the abbreviation of the selection antibiotic, and an isolate number if needed (e.g., S10CTX: isolate selected on cefotaxim from animal number 10 in the region South).

Based on multilocus sequence typing (MLST) analysis using the Achtman sequence typing scheme, 29 unique STs were identified among the 102 ESC-resistant E. coli isolates (Fig. 3; Table S3). The most frequently found STs were from the ST10 complex (ST-10 and ST-48 [12.5%; *n* = 13 each] and few other single-locus variants) corresponding to the phylogroup A, which are distributed in 16 farms of all regions. ST-117 was also prevalent and corresponded to all isolates of the phylogroup G (12.5%; *n* = 13). Other relevant STs known to be poultry associated (ST-93, ST-115, ST-189, ST-354, ST-457, and ST-1011) were represented by 4 to 8 isolates (Fig. 3; Table S3).

*In silico* serotyping analysis returned 40 different serotypes among 88 isolates. The O serotype was nontypeable for the 16 remaining isolates. The most predominant serotypes were O21:H16 (*n* = 8) and O11:H25 (*n* = 7). In this collection, isolates from the same serotype were found clustered in the phylogenetic single nucleotide polymorphism (SNP) analysis; however, there was no strict link between serotypes and phylogroups/ST except for the serogroup O11:H25 corresponding to all ST-457 isolates. Interestingly, the flagellar antigens H4, H9, H16, and H32 were more frequent than the others. The predominant poultry-associated ST-115 and ST-117 isolates were O-serotype diverse, but all harbored the flagellar antigens H9 and H4, respectively. Ten isolates belonged to known avian pathogenic-associated serotypes (O2, O5, O8, and O78; *n* = 2, 5, 2, and 1, respectively).

### Phylogenetic SNP analysis.

A phylogenetic tree was inferred from the core 176,818 SNP positions shared between all isolates. The phylogenetic tree grouped the 102 E. coli *sensu lato* isolates into 7 well-supported clusters consistent with their phylogroup distribution (Fig. 3). The two isolates S5CTX and S10CTX belonging to clade I clearly branched together and outside the 6 other groups corresponding to phylogroups A, B1, D, E, F, and G. Isolates exhibiting the same STs or ST complexes grouped closely in the phylogenetic SNP tree (Fig. 3).

In several STs, few highly related clonal groups of isolates showed a very small number of SNP differences (<40 SNPs in clusters of 9, 8, 5 and 5 isolates of ST-10, ST-48, ST-93, and ST-457, respectively). Although some isolates within these clonal groups originate from the same farm, consistent with strain diffusion in broiler flocks, all groups contained isolates from distinct farms, sometimes from distant regions (i.e., ST-10, ST-93, and ST-457 clonal groups). Such scattered distribution indicates that these well-known and poultry-associated clonal populations are spreading in the entire Lebanese poultry production. Beyond these clonal spreads, the geographic origin of the isolates at the regional and farm levels is distributed in the entire phylogenetic tree, indicating a wide spread of genetically diverse ESC-resistant isolates across the Lebanese territory.

### Acquired resistome analysis.

Acquired resistance genes and point mutations were detected using the tool ResFinder v2.1. Among the 102 ESC-resistant E. coli draft genomes, the detection of ESBL/AmpC and other β-lactamase genes was in agreement with the β-lactam resistance phenotypes (Table S2) defined earlier. Sixty isolates showing only ESBL phenotype (57%) carried one ESBL gene alone (*n* = 53) or two ESBL genes (*n* = 7) (Fig. 3; Table S2 and Table S3). All the isolates with an AmpC phenotype alone (*n* = 28) or in combination with an ESBL phenotype (*n* = 14) contained the AmpC cephalosporinase gene *bla*_CMY-2_. In addition, the narrow-spectrum β-lactamase *bla*_TEM-1_ variants were largely distributed and were found in 71 isolates (Table S3). The main ESBL genes were *bla*_CTX-M-3_ (31/102), *bla*_CTX-M-14/14b_ (9/102), *bla*_CTX-M-15_ (15/102), and *bla*_CTX-M-27_ (9/102). In addition, 8 isolates contained a couple of ESBL genes (*bla*_CTX-M-15_/*bla*_TEM-57_ or *bla*_CTX-M-3_/*bla*_TEM-57_ or *bla*_SHV-12_/*bla*_TEM-176_). Only one isolate carried the *bla*_CTX-M-1_ gene known to be livestock associated. It is noteworthy that one isolate, S4CTX, harbored 4 different β-lactam resistance genes (i.e., the ESBL genes *bla*_SHV-12_ and *bla*_TEM-176_, the AmpC cephalosporinase *bla*_CMY-2_, and the narrow-spectrum *bla*_TEM-1B_).

Overall, these 102 ESC-resistant E. coli isolates were multidrug-resistant (MDR), harboring different additional non-β-lactam resistance genes against aminoglycosides, phenicols, sulfonamides, trimethoprim, tetracycline, colistin, and fluoroquinolones (Table S3). A high diversity of non-β-lactam resistance gene patterns was observed among isolates that shared the same ESBL/AmpC resistance genes. The most predominant non-β-lactam resistance genes were the sulfonamide resistance genes (*sul1*, *sul2*, and *sul3*), the tetracycline resistance gene *tetA*, the florfenicol/chloramphenicol resistance gene *floR*, and the aminoglycoside resistance genes [*aadA1*, *aadA2*, *aac(3)-IId*, *aph(3′)-Ia*, and *strAB*].

Regarding other critically important resistance, nearly all 102 ESC-resistant isolates were ciprofloxacin resistant with a majority harboring at least the 3 QRDR mutations at positions 83 and 87 of DNA gyrase subunit A (GyrA) and position 80 of DNA topoisomerase IV subunit A (ParC) (data not shown). Moreover, the plasmid-mediated quinolone resistance genes *qnrB19*, *qnrD*, and *qnrS1* were found in 11, 1, and 10 isolates, respectively (Fig. 3). Another critically important resistance gene detected was the mobile colistin resistance gene *mcr*-*1* (Table S3). Nineteen ESBL/AmpC-producing E. coli isolates were positive for *mcr-1* (Fig. 3; Table S3). No specific cooccurrence of the *mcr*-*1* gene was found with any ESBL/AmpC gene nor with any phylogroup/ST/serotype or other non-β-lactam resistance gene.

The analysis of the plasmidic replicon content revealed a large diversity of replicon types among the 102 ESC-resistant E. coli isolates (Table S3). The isolates carried from only one up to seven replicon families (i.e., strain M40FEP was only IncF positive, and strain B23CTX was positive for Col, IncF, IncB/O/K/Z, IncHI, IncI, IncN, and IncX). IncF and Col replicons, which are well-known to be associated with E. coli, were present in nearly all isolates. Other Inc replicon groups, such as IncK, IncHI2, IncI2, and IncX4, were also detected among these ESC-resistant isolates. No specific cooccurrence of the main ESBL/AmpC genes (e.g., *bla*_CTX-M-3_) was found with any Inc groups. In addition, the repetitive nature of flanking regions of AMR genes and short-read assemblies did not allow for colocalization of the ESBL/AmpC genes and Inc replicons on identical contigs. On the other hand, the *mcr-1* gene was found located on IncI2 or IncX4 contigs in some isolates (data not shown). Further work is needed to determine precisely the genetic supports of these AMR genes.

### Phylogeographic distribution of E. coli carrying the main ESBL/AmpC and *mcr-1* genes.

**FIG 4:**
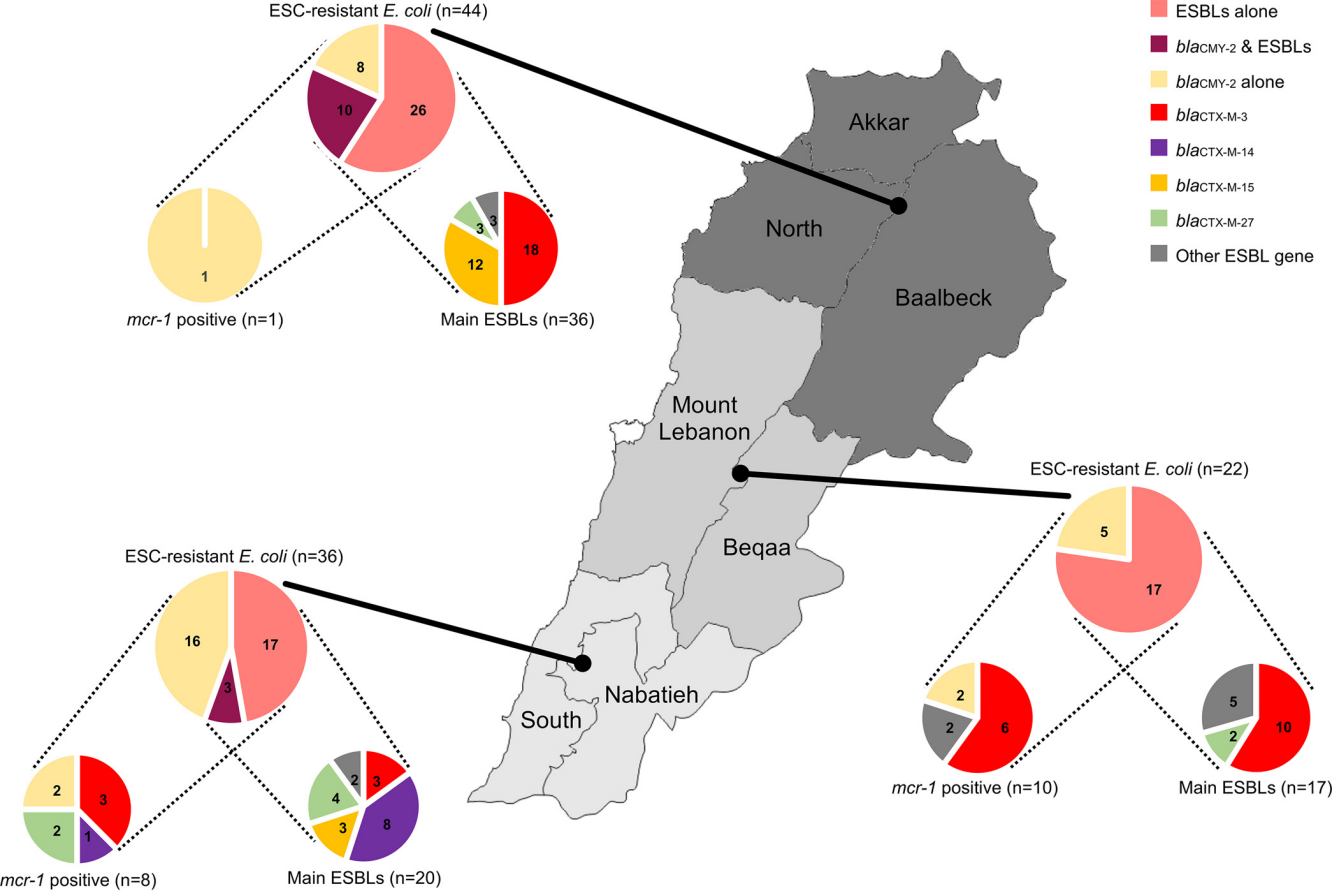
Regional distribution of *bla*_CMY-2_ and the main ESBL genes and their cooccurrence with the mobile colistin resistance gene *mcr-1*. In this figure, the Lebanese territory was divided into 3 geographical areas based on (i) similar sampled farm numbers (i.e., the North: North, Akkar, and Baalbeck administrative districts [20 farms]; the center: Mount Lebanon and Beqaa [17 farms]; and the South: South and Nabatieh [19 farms]) and on (ii) the distribution area of the major suppliers of 1-day-old chicks in Lebanon. Diagrams are based on ESBL/AmpC/*mcr-1* detection in the 102 E. coli draft genomes of this study. Diagrams of main ESBLs represent the subset of E. coli isolates carrying ESBLs alone or ESBLs and pAmpC CMY-2. Diagrams of *mcr*-*1*-positive isolates are based on all ESC-resistant E. coli for each region.

Here, the Lebanese territory was divided in 3 geographical areas based on similar sampled farm numbers, that is, the North (20 farms; North, Akkar, and Baalbeck administrative districts), the center (17 farms; Mount Lebanon and Beqaa), and the South (19 farms; South and Nabatieh) (Fig. 4). The North and the South showed the highest and lowest numbers of ESC-resistant E. coli (*n* = 129 and 55; Table S2), respectively.

Interestingly, the distribution of the ESBL/AmpC genes and the *mcr-1* gene displayed some specificities between these 3 geographical regions (Fig. 4). The ESBL gene *bla*_CTX-M-3_ was predominant in the North and the center, representing around half of the ESBL genes detected, while the South displayed primarily the *bla*_CTX-M-14/14b_ gene absent from the above regions. Whereas *bla*_CTX-M-15_ represented one-third of the ESBL gene in the North region, it is absent in the center. Interestingly, the geographic predominance of *bla*_CTX-M-14/14b_ and *bla*_CTX-M-15_ in the South and North is mainly driven by the spread of clonal groups of ST-48 (<30 SNPs) and ST-10 (<23 SNPs) in different farms of these regions, respectively. Other ESBL/AmpC genes are punctually distributed across the phylogenetic tree, farms, and regions, suggesting that they are probably not widely disseminated in the poultry E. coli population (Fig. 3).

The presence of the colistin resistance gene *mcr-1* in ESBL/AmpC-producing E. coli is mainly found in center and South Lebanon but in different unrelated clusters of isolates distributed in the phylogenetic tree and in different farms (Fig. 3 and 4). Among the 19 *mcr-1*-positive ESBL/AmpC-producing E. coli, the occurrence of *mcr-1* appeared also independent of a specific ESBL/AmpC gene. Together, all these results strongly suggest that these critically important resistance genes are likely located on different mobile genetic supports that can be acquired independently by different isolates and that participated in their dissemination at various geographic scales. In some cases (i.e., *bla*_CTX-M-14/14b_ ST-48 and *bla*_CTX-M-15_ ST-10), the regional spread of clonal populations also participates in the diffusion of these resistances. Finally, the multiple component analysis (MCA) of these E. coli genomic data regarding farm information did not return any relevant explicative factor for the selection or dissemination of these resistances relative to breeding practices.

### Virulence-associated gene content of the main poultry-associated STs.

Fifty-two genes encoding virulence factors were detected among the 102 E. coli collection using the Virulence Finder tool v2.0 (Table S3). APEC strains are known to harbor different associations of virulence-associated genes (VAGs), constituting a heterogeneous group of extraintestinal pathogenic E. coli in terms of virulence factors. Isolates belonging to phylogroups A, B1, and E harbored less VAGs than other phylogroups D, F, and G (Fig. S2 and S3A; *P* < 0.0001). ST-93 isolates belonging to phylogroup A harbored 13 to 14 VAGs, including different agglutinins/adhesion factors, iron acquisition systems, and capsule and colicin biosynthesis. Among the phylogroup A isolates, closely related isolates of the clonal group ST-10 (*n* = 9, <23 SNPs) as well as two ST-189 isolates harbor the intimin Eae and the type III secretion system and its effectors (EspABFJ, NleB, and Tir), which are implicated in adhesion and enterocyte effacement, respectively. In phylogroups D, F, and G, which gathered the typical poultry-associated STs (ST-115, ST-117, ST-354, and ST-457), numerous isolates harbored VAGs encoding adhesion factors, in particular type P fimbriae or long polar fimbriae, several siderophores (yersiniabactin, salmochelin, and aerobactin), capsule biosynthesis, and colicin/microcin biosynthesis. In this regard, ST-115 and ST-117 isolates harbored the highest VAG content compared to other major STs (mean of >18 VAGs; Fig. S3B). All these VAGs are common in APEC and other extraintestinal pathogenic E. coli lineages and play an important role in colonization of different niches.

## DISCUSSION

Despite the implementation of interventional measures to prevent the spread of antibiotic resistance in developed countries, this issue is still a growing problem worldwide. In developing countries such as Lebanon, the resistance crisis is more complex, since numerous factors concerning the dissemination of antibiotic resistance remain uncontrollable. In the one health context of antibiotic resistance, livestock is now considered a major reservoir of multidrug-resistant bacteria and antibiotic resistance genes. While numerous studies have targeted antibiotic resistance in human clinical settings in Lebanon, the epidemiological situation in food-producing animals remains unclear (8). In the present study, we report an alarming intestinal carriage of ESC-resistant *Enterobacterales* in broilers farms at the national level. ESC-resistant isolates were detected in 78.5% of the collected samples. Fifty-two of 56 farms (93%) housed broilers carrying ESC-resistant isolates that are also resistant to many other antibiotics. In a previous study conducted in 2015, Dandachi et al. reported only 20.6% of broiler fecal samples carrying ESC-resistant *Enterobacterales*, and 77.5% of the visited farms had at least one positive animals (10). Taken together, these results suggest an increase of ESC-resistant isolate carriage in poultry in Lebanon during the last few years. The prevalence found in the present study is among the highest described in comparable epidemiological studies of resistance in poultry worldwide (17).

ESC-resistant Escherichia coli was the most common ESC-resistant species isolated, representing more than 87% (Table 2; see also Table S2 in the supplemental material). Moreover, all ESC-resistant E. coli isolates were considered multidrug resistant (≥3 antimicrobial classes) but remained susceptible to carbapenems. These isolates showed mainly ESBL (71%), AmpC (11%), or both phenotypes. During the sample collection, we collected only generic data of antibiotic administration (used antibiotics, treatment duration, and causes of administration) as it was impossible for most farms to obtain precise information on the amount of antibiotics, the frequency of administration, and effective reasons of usage (treatment or prevention of infections and growth enhancement). Depending on the farm, sampled flocks were treated with a variety of antibiotic regimens, ranging from one antibiotic to a large cocktail of antibiotics (Table 1). Doxycycline, colistin, gentamicin, and ciprofloxacin were the most used antibiotics in the sampled flocks, indicating that they are frequently used in poultry farms in Lebanon (Fig. S1). Last-generation cephalosporins were only used in 8.9% of farms, but other β-lactam antibiotics, such as amoxicillin, ampicillin, and cephalexin, were more frequently administered and generated selection pressures favorable to ESC-resistant isolates. These antibiotic uses are in accordance with a recent evaluation of availability of colistin-containing drugs for poultry and antibiotic residues in chicken meat in Lebanon (18, 19).

Genomic analysis of these ESBL/AmpC-producing E. coli revealed a high diversity of isolates in poultry farms across the Lebanese territory. Only a few highly related clonal groups (<40 SNPs) of known poultry-associated STs (ST-10, ST-48, and ST-93) are spreading in the entire Lebanese poultry production (Fig. 3). This current observation is slightly different from the previous similar nationwide study carried out in 2015 that described a regional spreading and the absence of known poultry-associated STs in Lebanese poultry production (10). While being isolated from healthy animals, some of these ESBL/AmpC-producing E. coli isolates belonged to avian pathogenic-associated STs (ST-115, ST-117, ST-354, and ST-457) and harbored a large content of virulence-associated genes, suggesting a pathogenic potential (7, 20).

In the present study, the plasmidic AmpC cephalosporinase gene *bla*_CMY-2_ was the most prevalent ESC resistance gene (40% positive E. coli isolates) and appears to be maintained at a high level in the Lebanese poultry production since 2015 (10). In addition, the most predominant ESBL enzymes were the CTX-M enzymes, consistent to what is described worldwide (3, 4, 21). Several studies showed that the predominant ESBL genotypes identified in broiler E. coli isolates were either the *bla*_CTX-M-1_ gene, which is usually related to animals, or the *bla*_CTX-M-15_ gene, which is also frequently reported in humans (4, 21). Surprisingly, only one isolate in our survey contained the *bla*_CTX-M-1_ gene, and the majority of *bla*_CTX-M-15_-positive isolates corresponded to a unique highly related clonal ST10 group. The dominant ESBL gene was *bla*_CTX-M-3_, which is spreading in genetically diverse E. coli in the entire Lebanese poultry production. To our knowledge, this represents the first description of the *bla*_CTX-M-3_ gene in Lebanon. Moreover, it is worth noting that this gene has been rarely described in poultry. Since its first description in 1995, the *bla*_CTX-M-3_ gene has been dominant in humans in Poland in the early 2000s and sporadically disseminated on an IncM2 plasmid (pCTX-M-3) to southern Europe and Turkey (3, 4, 21). Nowadays, CTX-M-3 remains at an elevated proportion in Polish, Russian, and Greek populations (4, 21). Recently, pCTX-M3 has also been described in different Salmonella enterica serovars and other *Enterobacterales* species of hospitalized horses in Israel, suggesting that the spread of this plasmid in this part of the Middle East may have regional specificity (22). Other CTX-M types (CTX-M-14, CTX-M-27, and CTX-M-55) were also significantly detected as previously described in various human, animal, and environmental settings in Lebanon (2).

In addition, almost all ESC-resistant *Enterobacterales* isolates in this study were also ciprofloxacin resistant (Fig. 2). This result was confirmed by the genomic analysis of 102 E. coli isolates showing at least the 3 QRDR mutations in GyrA and ParC known to confer high-level resistance to fluoroquinolones. Finally, we report an important cooccurrence (>18%) of the mobile colistin resistance gene *mcr-1* in ESBL/AmpC-producing E. coli in poultry. In Lebanon, the *mcr-1* gene has been recently reported in poultry, swine, aquaculture, and humans but rarely in ESC-resistant isolates (11–13, 15, 23, 24). In a recently published study focusing on colistin-resistant E. coli from poultry at a slaughterhouse in Lebanon, the same cooccurrence of the mobile colistin resistance gene *mcr-1* and ESBL/AmpC genes has been described (24). While common *mcr-1*-positive E. coli STs (i.e., ST1011, ST744, and ST48) have been isolated in their study and ours, different associations with ESBL/AmpC-encoding genes are found, strengthening the hypothesis of the dissemination of these critical resistance genes by different plasmids. All together, these results highlight the occurrence of extremely drug resistant (XDR) E. coli isolates (i.e., resistant to last-generation cephalosporins, fluoroquinolone, and colistin) in Lebanese broiler production. Such XDR isolates, some of them belonging to E. coli STs known to be zoonotic (ST-48, ST-93) or harboring important virulence-associated genes (Fig. S2), sometimes are associated with extraintestinal human infections and may pose therapeutic challenges if transmission to humans occurs.

In conclusion, the present study illustrates the current genomic epidemiology of multidrug-resistant *Enterobacterales* in Lebanese broiler farms. ESBL and AmpC producers are highly prevalent across the territory. In addition, these ESBL/AmpC isolates are also resistant to other medically important antibiotics (i.e., colistin and ciprofloxacin). Their complex genomic epidemiology highlighted by an important genetic diversity suggests that diverse mobile genetic elements, probably conjugative plasmids carrying these resistance genes, are largely spreading in enteric bacteria in the Lebanese poultry production. Further studies are needed in order to characterize precisely AMR plasmids to understand their specific epidemiology. The real purposes (treatment, prophylaxis, or growth promotion) and precise levels of antibiotic use remain unclear and difficult to get from poultry farm owners and veterinarians, which may have hindered our capacity to link the antibiotic resistance level to antibiotic usage at the farm level. Further surveillance programs and decisive interventions are urgently needed in order to ban the use of critically important antibiotics for human medicine in food-producing animals and limit the spread of antibiotic resistance in Lebanon.

## MATERIALS AND METHODS

### Sampling, bacterial isolates, and antimicrobial susceptibility testing.

Cloacal swabs were collected from 280 individual broilers during summer and fall 2018 in 56 fattening farms (5 broilers per farm) in the seven main regions of Lebanon (South, Nabatieh, North, Akkar, Beqaa, Baalbeck, and Mount Lebanon; see Table S1 in the supplemental material). A questionnaire has been completed by the veterinarian of each farm regarding the chickens’ diet, intake of food supplements such as vitamins, and antibiotics administration (classes of antibiotics, quantity, and causes of administration). Swabs were incubated in peptone water broth at 37°C overnight. ESC-resistant *Enterobacterales* isolates were selected on MacConkey agar plates supplemented with either cefotaxime (2 mg/liter) or cefepime (4 mg/liter). Species identification was done using the API 20E assay (bioMérieux, Marcy-l'Étoile, France). To avoid strain redundancy, only one strain of each species per selection was conserved at −80°C in 15% glycerol brain heart infusion (BHI) broth for further analysis. Each isolate was tested for antimicrobial susceptibility by the disk diffusion method on Mueller-Hinton agar as recommended by EUCAST 2018 (http://www.eucast.org/). Susceptibility to a panel of 13 antibiotics and the production of ESBL using the double disk synergy test were determined using disks containing the following antibiotics in accordance with EUCAST 2018 clinical breakpoint: amoxicillin/clavulanate (20/10 μg), cefoxitin (30 μg), cefotaxime (5 μg), ceftazidime (10 μg), cefepime (30 μg), imipenem (10 μg), ertapenem (10 μg), aztreonam (30 μg), gentamicin (10 μg), amikacin (30 μg), ciprofloxacin (5 μg), tetracycline (30 μg), and trimethoprim-sulfamethoxazole (1.25/23.75 μg).

### Whole-genome sequence analysis.

A set of 102 E. coli isolates representative of the diversity of antimicrobial resistance profiles and origins was subjected to whole-genome sequencing. Briefly, DNA extraction was performed using a NucleoSpin 8 tissue kit (Macherey-Nagel, Düren, Germany), and DNA samples were sent to Novogene Co., Ltd., for library preparation using the New England BioLabs Next Ultra DNA library prep kit and whole-genome sequencing on NovaSeq (Illumina) using 2 × 150-bp paired-end sequencing (Novogene, Beijing, China).

The paired-end, 150-bp-long raw reads were quality filtered, and Illumina adapters were removed by Novogene before data delivery, resulting in read quality above 30 at all positions. After a second quality checking using fastQC v0.11.7 (https://www.bioinformatics.babraham.ac.uk/projects/fastqc/), reads were assembled using SPAdes v3.13.0 with the –only assembler option (25). Since expected read coverage was 60× to 100×, assembled scaffolds with an estimated coverage at least twice as low (<30×) were discarded as well as those shorter than 200 bp. Sample contamination control was finally achieved using CheckM with the genus Escherichia and the –skip_pseudogene_correction options (26).

All 102 genomes were submitted to the Bacterial Analysis Pipeline v1.1 for *in silico* sequence type analysis with the MLST tool v1.6 for antibiotic resistance gene detection with the ResFinder tool v2.1 using default parameters and for replicon type detection with the PlasmidFinder tool v2.1 using default parameters. The genomes were then submitted individually to the SerotypeFinder tool v.2 and the VirulenceFinder tool v.2 using default parameters for serotype and virulence gene detection, respectively (27–30).

The phylogroup assignation was determined with Clermontyping v1.4.1 (31). This tool uses two different algorithms to infer the phylogroup: an *in silico* PCR method based on the presence/absence of 5 loci and a method based on the whole-genome similarity to reference strains. In case of discordant assignation, the genome-based output was preferred, except for five genomes (A14FEP [CAJPVB000000000], ND35FEP [CAJPXD000000000], S24CTX [CAJPYG000000000], S31CTX [CAJPYK000000000], and S32CTX [CAJPYQ000000000]). These strains were all assigned to phylogroup G by the *in silico* PCR method and to phylogroup F with the whole-genome method due to a reference genome incorrectly assigned in this tool at the time of the study (E. coli 327_20; O. Clermont, personal communication).

A phylogenetic tree based on genome-wide single nucleotide polymorphisms (SNPs) was produced using the parSNP tool, which is based on FastTree 2 maximum likelihood phylogenetic reconstruction (32, 33). The software was run with the Escherichia coli K-12 MG1655 genome (GenBank accession U00096) as a reference for SNP calling and other parameters as default. The phylogenetic tree was visualized and annotated with the iTOL web interface tool v4 (34).

### Statistical analysis.

The analysis of the phenotypic and genomic data sets was conducted in Python using the Pandas 1.1.0 library for data analysis (https://pypi.org/project/pandas/) and the Prince 0.7 library for factor analysis (https://pypi.org/project/prince/): principal-component analysis (PCA) for numeric variables, multiple component analysis (MCA) for categorical variables, and factor analysis of mixed data (FAMD) for combinations of numeric and categorical variables. The “n_iter” parameter of Prince analyses was set to 3 as recommended as it converges quickly.

At the phenotypic level, the 341 isolates were studied for the association between their antibiotic resistance profiles and the explanatory variables of the questionnaire, including antibiotic administration, purpose of antibiotic usage, diet, nutrition supplements, region, and farm. Antibiotic resistance profiles were created as Pandas categorical data frames (resistant or susceptible) and fitted to MCA. At the genomic level, the 102 E. coli isolates were studied for the association between the resistance genes present in the genomes and the explanatory variables of the questionnaire as listed above plus their sequence type (ST).

### Data availability.

Genome assemblies can be accessed through the European Nucleotide Archive (ENA) database under project accession number PRJEB43674. Strain ENA accession numbers are listed in Table S3.
