# Supplementary material for: Occurrence of the Colistin Resistance Gene mcr-1 and Additional Antibiotic Resistance Genes in ESBL/AmpC-Producing Escherichia coli from Poultry in Lebanon: A Nationwide Survey
Source: Microbiol Spectr. 2021 Sep 8;9(2):e00025-21. doi: 10.1128/Spectrum.00025-21 (PMC8557922; doi:10.1128/Spectrum.00025-21)

Figure S1. Proportion of broiler farms using each antibiotic.
DOX: Doxycycline, CST: Colistin, GEN: Gentamicin, CIP: Cirpofloxacin, AMX: Amoxicillin, SXT: Trimethoprim+Sulfamethoxazole, TLM: Tilmicosin, LEX: Cefalexin, FLO: Florfenicol, TYL: Tylosin, CPD: Cefpodoxime, SPM: Spyramicin, ENR: Enrofloxacin, ERY: Erythromycin, NEO: Neomycin, SDZ: Sulfadiazin, TET: Tetracycline.

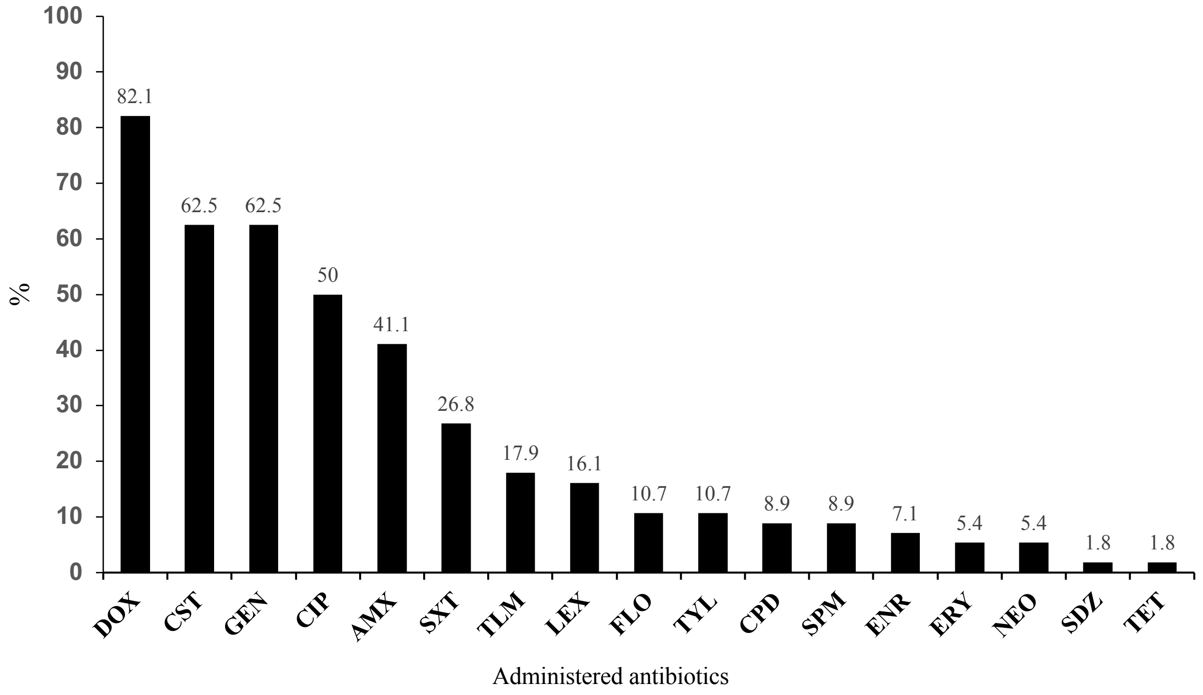

Figure S2. Phylogenetic relationships between the 102 sequenced ESC-resistant *E. coli* strains. The tree was inferred from the 176,818 SNPs present in genomic regions shared by all isolates. Local branching support values are based on the Shimodaira-Hasegawa test. Values of 1, indicating maximum support, are not displayed. The tree was rooted according to the known relationships between *E. coli* phylogroups. For each strain, the sequence type, serotype, identifier, region and farm of origin are indicated in columns 1-5 on the right side of the tree. Presence of virulence-associated genes detected using VirulenceFinder 2.0 are indicated with a filled box.

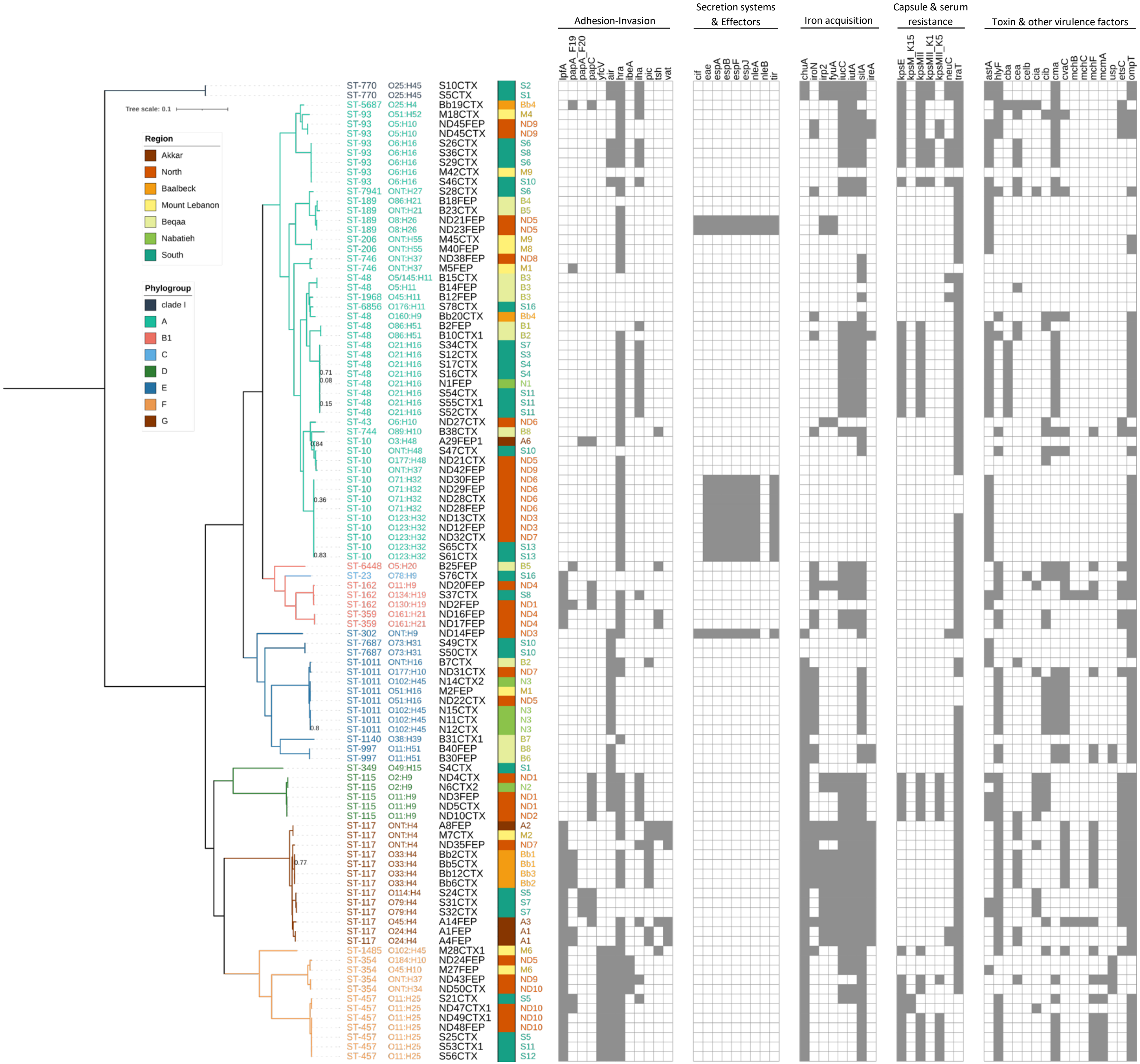

Adhesion-Invasion
Secretion systems & Effectors
Iron acquisition
Capsule & serum resistance
Toxin & other virulence factors
lpfA papA_F19 papA_F20 papC yfcV air hra ibeA iha pic tsh vat
cif eae espA espB espF espJ nleA nleB tir
chuA iroN irp2 fyuA iucC iutA sitA ireA
kpsE kpsM_K15 kpsMII kpsMII_K1 kpsMII_K5 neuC traT
astA hlyF cba cea celb cia cib cma cvaC mchB mchC mchF mcmA usp etsC ompT
Tree scale: 0.1
Region
Akkar
North
Baalbeck
Mount Lebanon
Beqaa
Nabatieh
South
Phylogroup
clade I
A
B1
C
D
E
F
G
0.71
0.08
0.15
0.84
0.36
0.83
0.8
0.77
ST-770 O25:H45 S10CTX S2
ST-770 O25:H45 S5CTX S1
ST-5687 O25:H4 Bb19CTX Bb4
ST-93 O51:H52 M18CTX M4
ST-93 O5:H10 ND45FEP ND9
ST-93 O5:H10 ND45CTX ND9
ST-93 O6:H16 S26CTX S6
ST-93 O6:H16 S36CTX S8
ST-93 O6:H16 S29CTX S6
ST-93 O6:H16 M42CTX M9
ST-93 O6:H16 S46CTX S10
ST-7941 ONT:H27 S28CTX S6
ST-189 O86:H21 B18FEP B4
ST-189 ONT:H21 B23CTX B5
ST-189 O8:H26 ND21FEP ND5
ST-189 O8:H26 ND23FEP ND5
ST-206 ONT:H55 M45CTX M9
ST-206 ONT:H55 M40FEP M8
ST-746 ONT:H37 ND38FEP ND8
ST-746 ONT:H37 M5FEP M1
ST-48 O5/145:H11 B15CTX B3
ST-48 O5:H11 B14FEP B3
ST-1968 O45:H11 B12FEP B3
ST-6856 O176:H11 S78CTX S16
ST-48 O160:H9 Bb20CTX Bb4
ST-48 O86:H51 B2FEP B1
ST-48 O86:H51 B10CTX1 B2
ST-48 O21:H16 S34CTX S7
ST-48 O21:H16 S12CTX S3
ST-48 O21:H16 S17CTX S4
ST-48 O21:H16 S16CTX S4
ST-48 O21:H16 N1FEP N1
ST-48 O21:H16 S54CTX S11
ST-48 O21:H16 S55CTX1 S11
ST-48 O21:H16 S52CTX S11
ST-43 O6:H10 ND27CTX ND6
ST-744 O89:H10 B38CTX B8
ST-10 O3:H48 A29FEP1 A6
ST-10 ONT:H48 S47CTX S10
ST-10 O177:H48 ND21CTX ND5
ST-10 ONT:H37 ND42FEP ND9
ST-10 O71:H32 ND30FEP ND6
ST-10 O71:H32 ND29FEP ND6
ST-10 O71:H32 ND28CTX ND6
ST-10 O71:H32 ND28FEP ND6
ST-10 O123:H32 ND13CTX ND3
ST-10 O123:H32 ND12FEP ND3
ST-10 O123:H32 ND32CTX ND7
ST-10 O123:H32 S65CTX S13
ST-10 O123:H32 S61CTX S13
ST-6448 O5:H20 B25FEP B5
ST-23 O78:H9 S76CTX S16
ST-162 O11:H9 ND20FEP ND4
ST-162 O134:H19 S37CTX S8
ST-162 O130:H19 ND2FEP ND1
ST-359 O161:H21 ND16FEP ND4
ST-359 O161:H21 ND17FEP ND4
ST-302 ONT:H9 ND14FEP ND3
ST-7687 O73:H31 S49CTX S10
ST-7687 O73:H31 S50CTX S10
ST-1011 ONT:H16 B7CTX B2
ST-1011 O177:H10 ND31CTX ND7
ST-1011 O102:H45 N14CTX2 N3
ST-1011 O51:H16 M2FEP M1
ST-1011 O51:H16 ND22CTX ND5
ST-1011 O102:H45 N15CTX N3
ST-1011 O102:H45 N11CTX N3
ST-1011 O102:H45 N12CTX N3
ST-1140 O38:H39 B31CTX1 B7
ST-997 O11:H51 B40FEP B8
ST-997 O11:H51 B30FEP B6
ST-349 O49:H15 S4CTX S1
ST-115 O2:H9 ND4CTX ND1
ST-115 O2:H9 N6CTX2 N2
ST-115 O11:H9 ND3FEP ND1
ST-115 O11:H9 ND5CTX ND1
ST-115 O11:H9 ND10CTX ND2
ST-117 ONT:H4 A8FEP A2
ST-117 ONT:H4 M7CTX M2
ST-117 ONT:H4 ND35FEP ND7
ST-117 O33:H4 Bb2CTX Bb1
ST-117 O33:H4 Bb5CTX Bb1
ST-117 O33:H4 Bb12CTX Bb3
ST-117 O33:H4 Bb6CTX Bb2
ST-117 O114:H4 S24CTX S5
ST-117 O79:H4 S31CTX S7
ST-117 O79:H4 S32CTX S7
ST-117 O45:H4 A14FEP A3
ST-117 O24:H4 A1FEP A1
ST-117 O24:H4 A4FEP A1
ST-1485 O102:H45 M28CTX1 M6
ST-354 O184:H10 ND24FEP ND5
ST-354 O45:H10 M27FEP M6
ST-354 ONT:H37 ND43FEP ND9
ST-354 ONT:H34 ND50CTX ND10
ST-457 O11:H25 S21CTX S5
ST-457 O11:H25 ND47CTX1 ND10
ST-457 O11:H25 ND49CTX1 ND10
ST-457 O11:H25 ND48FEP ND10
ST-457 O11:H25 S25CTX S5
ST-457 O11:H25 S53CTX1 S11
ST-457 O11:H25 S56CTX S12

Figure S3. Virulence-associated genes by *E. coli* phylogroup (A) or main STs (B). Means and SD are represented (two-way ANOVA test, statistical significance is indicated as follow: ****, $P<0.0001$; ***, $P<0.001$).

A.

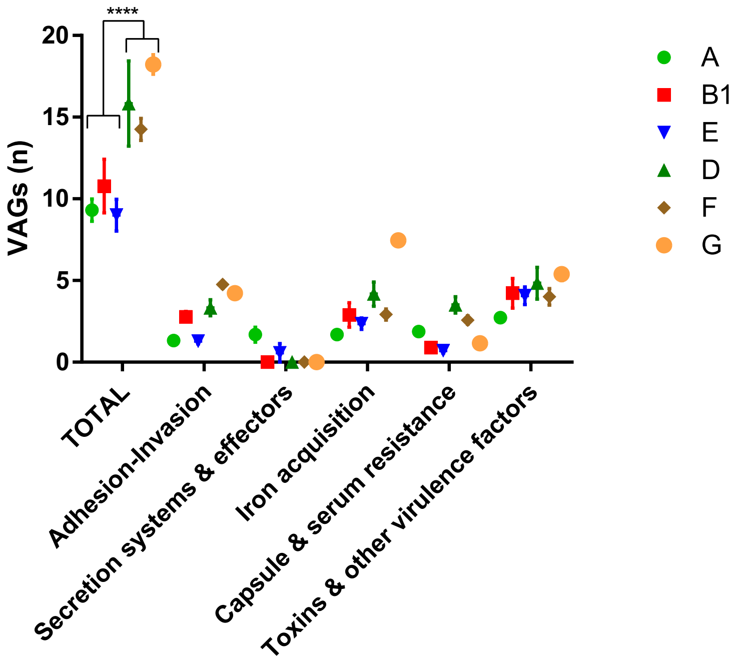

B.

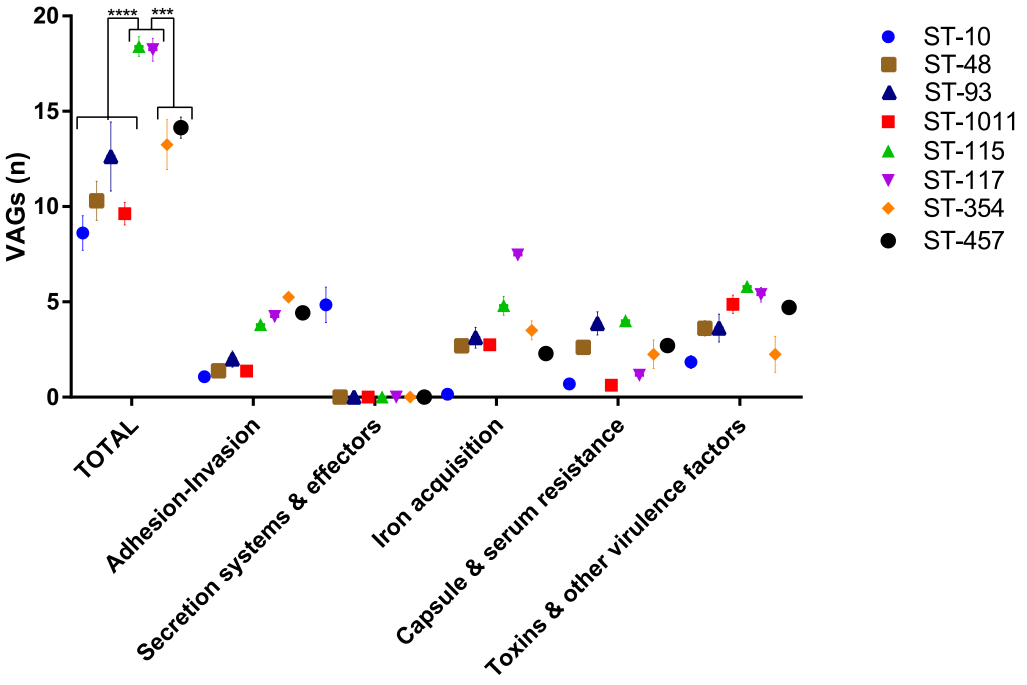

Supplement: SUPPLEMENTAL FILE 1 — Supplemental material. Download Spectrum.00025-21-s0001.pdf, PDF file, 0.9 MB [file spectrum.00025-21-s0001.pdf]
